# Protocol for metabolic profiling of antigen-specific CD8^+^ T cells using spectral flow cytometry

**DOI:** 10.1016/j.xpro.2026.104517

**Published:** 2026-04-22

**Authors:** J. Fréderique de Graaf, Nils Mülling, Ramon Arens

**Affiliations:** 1 Department of Immunology, Leiden University Medical Center, Leiden 2333 ZA, the Netherlands; 2 Department of Infectious Disease and Nephrology, University Hospital Essen, University Duisburg-Essen, 45147 Essen, Germany

**Keywords:** Cell Biology, Immunology, Metabolism

## Abstract

CD8^+^ T cells rely on tightly regulated metabolic remodeling to support effector function. Here, we present a protocol for single-cell metabolic profiling of rare antigen-specific CD8^+^ T cells in unstimulated and antigen-stimulated conditions using spectral flow cytometry. The workflow enables detailed assessment of key metabolic pathways, including glycolysis, fatty acid oxidation, amino acid metabolism, the pentose phosphate pathway, and mitochondrial oxidative phosphorylation.

For complete details on the use and execution of this protocol, please refer to Mülling et al.^1^

## Highlights

• Step-by-step workflow for reproducible spectral flow metabolic profiling
• Stimulation of antigen-specific CD8^+^ T cells derived from blood or spleen
• Optimized antibody panels quantify key metabolic enzymes in resting/active states
• Guidance on analysis and interpretation of metabolic pathway activity in T cells

## Abstract (editor highlights)

Publisher’s note: Undertaking any experimental protocol requires adherence to local institutional guidelines for laboratory safety and ethics.

## Graphical abstract

**Figure:**
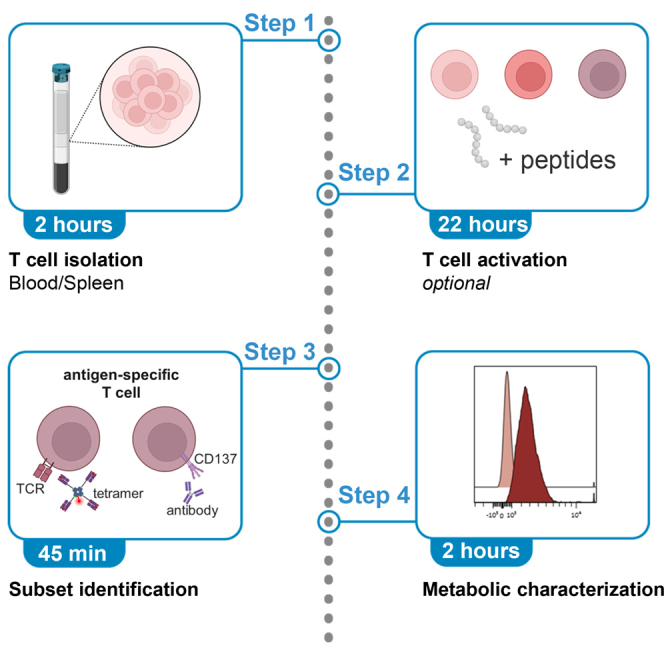

## Before you begin

**Figure 1:**
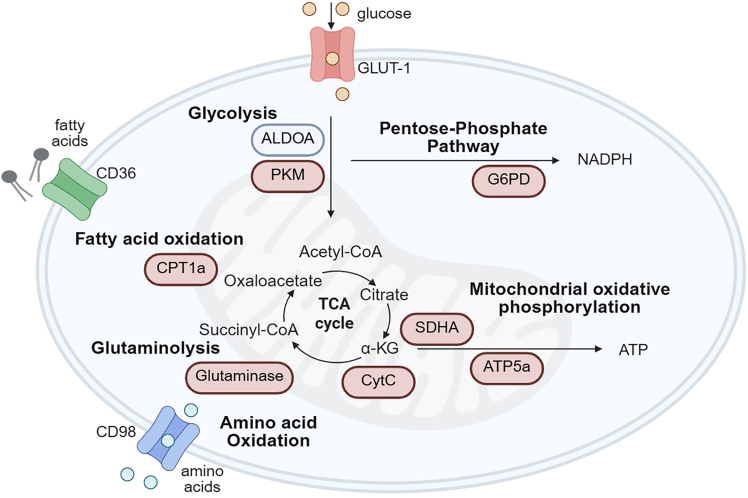
Schematic illustration of metabolic pathways and key enzymes quantified by flow cytometry in the described workflow The panel design focusses on Glycolysis (PKM, or alternatively ALDOA), Pentose-Phosphate Pathway (G6PD), Fatty Acid Oxidation (CPT1a), Glutaminolysis (GLS), Amino Acid Oxidation (CD98) and Mitochondrial Oxidative Phosphorylation (CytC, SDHA, ATP5a).

This protocol describes a spectral flow cytometry-based workflow for resolving metabolic features of rare human antigen-specific CD8^+^ T cells isolated from blood or spleen. The panel is designed to characterize and quantify the expression of key metabolic enzymes of major metabolic pathways, including glycolysis, fatty acid oxidation, amino acid metabolism (e.g., glutaminolysis), the pentose-phosphate pathway and mitochondrial oxidative phosphorylation, in antigen-specific T cell populations either unstimulated (resting) or following activation (Figure 1).

Before starting, ensure that your flow cytometer provides sufficient spectral resolution to discriminate the selected fluorochromes and that all antibodies and antibody panels have been validated on your specific instrument configuration. Additional examples of validated metabolic profiling panels for both murine and human samples are provided in Mülling *et al.*^1^

### Preparation of PMA and ionomycin stock and working solutions

**Timing: 10 min**

Phorbol 12-myristate 13-acetate (PMA) and ionomycin are commonly used together as a potent, non-specific cell stimulation cocktail for T cells. PMA acts as a diacylglycerol analog that directly activates protein kinase C, thereby bypassing membrane receptor signaling and triggering downstream pathways involved in cell activation. Ionomycin is a calcium ionophore that increases intracellular calcium levels by transporting Ca^2+^ across cellular membranes, complementing PMA-mediated signaling. In this workflow, the combination of PMA and ionomycin is used as positive non-specific control for antigen-specific peptide stimulation. Stock solutions are prepared under sterile conditions.

1. Dissolve 1 mg PMA and 1 mg calcium ionomycin separately in 1 mL DMSO to obtain stock solutions of 1 mg/mL.
2. Incubate for 10 min at room temperature (20°C) until the solution is clear.
3. Store aliquoted stocks at −80°C.
4. Prepare working stocks for PMA and calcium ionomycin:
  a. PMA (10 μg/mL): Dilute the 1 mg/mL PMA stock 100-fold with PBS to obtain a 10 μg/mL working stock.
  b. Calcium ionomycin (100 μg/mL): Dilute the 1 mg/mL calcium ionomycin stock 10-fold to obtain a 100 μg/mL working stock.

### Innovation

The growing interest in cellular metabolism, especially in chronic infection and cancer, highlights how metabolic dysregulation shapes CD8^+^ T cell impairment and motivates strategies to therapeutically reprogram T cell function. However, defining metabolic states of antigen-specific CD8^+^ T cells is often limited by their low frequency. This protocol illustrates how single-cell platforms, in particular spectral flow cytometry, can integrate antigen specificity with multidimensional metabolic profiling. The workflow is readily adaptable to other rare immune subsets, enabling metabolic characterization directly within heterogeneous, minimally processed samples.

### Institutional permissions

Buffy coats were obtained from healthy adult donors seropositive for CMV through voluntary donation at the Sanquin Blood Bank (Amsterdam, the Netherlands). Splenic tissue was collected from Dutch solid-organ transplant donors during explant procedures performed for routine HLA typing, in compliance with Dutch national legislation governing organ donation.

## Key resources table

**Table:** 

REAGENT or RESOURCE	SOURCE	IDENTIFIER
**Antibodies**		

HLA-A∗01 HCMV pp65_363-373_ (YSEHPTFTSQY) tetramer – APC (1:50 dilution)	LUMC	n.a.
HLA-A∗02 HCMV pp65_495-503_ (NLVPMVATV) tetramer – APC (1:50 dilution)	LUMC	n.a.
APC/Fire™ 810 anti-human CD8 Antibody (1:1600 dilution)	BD	Cat# 344764; RRID: AB_2860890
Brilliant Violet 711™ anti-human CD25 Antibody (1:200 dilution)	Biolegend	Cat# 356138; RRID: AB_2632781
Brilliant Violet 605™ anti-human CD69 Antibody (1:50 dilution)	Biolegend	Cat# 310938; RRID: AB_2562307
Brilliant Violet 650™ anti-human CD137 (4-1BB) Antibody (1:50 dilution)	Biolegend	Cat# 309828; RRID: AB_2572193
BD OptiBuild™ BUV496 Mouse Anti-Human CD45RA (1:1600 dilution)	BD Biosciences	Cat# 750258; RRID: AB_2874456
Brilliant Violet 785™ anti-human CD197 (CCR7) Antibody (1:50 dilution)	Biolegend	Cat# 353230; RRID: AB_2563630
BD Horizon™ BUV395 Mouse Anti-Human CD3 (1:50 dilution)	BD	Cat# 563548; RRID: AB_2744387
PE/Dazzle™ 594 anti-human Ki-67 Antibody (1:200 dilution)	Biolegend	Cat# 350533; RRID: AB_2566228
Anti-Glucose Transporter GLUT1 antibody [EPR3915] - BSA and Azide free (1:600 dilution)	Abcam	Cat# ab252403; RRID: AB_3719889
Anti-ATP5A antibody [EPR13030(B)] - BSA and Azide free (1:600 dilution)	Abcam	Cat# ab231692
Anti-PKM antibody [EPR10138(B)] - BSA and Azide free (1:400 dilution)	Abcam	Cat# ab206129, RRID: AB_3718102
Anti-SDHA antibody [EPR9043(B)] - BSA and Azide free (1:2000 dilution)	Abcam	Cat# ab240098
Anti-Glutaminase C antibody [EPR19525] - BSA and Azide free (1:600 dilution)	Abcam	Cat# ab223129
APC/Fire™ 810 anti-human CD38 Antibody (1:50 dilution)	Biolegend	Cat# 303549; RRID: AB_2860783
Anti-CPT1A antibody [EPR21843-71-2F] - BSA and Azide free (1:600 dilution)	Abcam	Cat# ab235841
Anti-Glucose 6 Phosphate Dehydrogenase antibody [EPR20668] - BSA and Azide free (1:800 dilution)	Abcam	Cat# ab231828
BD OptiBuild™ BUV395 Mouse Anti-Human CD98 (1:400 dilution)	BD	Cat# 744508; RRID AB_2742283
Anti-Cytochrome C antibody [7H8.2C12] - BSA and Azide free (1:600 dilution)	Abcam	Cat# ab237966

**Biological samples**		

Buffy coat	Sanquin	NA
Human Spleen	LUMC	NA

**Chemicals, peptides, and recombinant proteins**		

Penicilin, Streptomycin	Gibco	Cat# 15140-122
L-Glutamine	Gibco	Cat# 25030-024
IMDM	Gibco	Cat# 2478927
Human Serum	Gibco	Cat# 2580523
Fetal Calfs Serum (FCS)	Bodinco BV	Cat# BDC-S00FD1
DMSO	Merck	Cat# D5879-500ml
PBS	Fresenius Kabi	Cat# 16SD7330
Sodium Azide (10%)	Apotheek AZL	LUMC_97936383
Ficoll (5.7%) – Amidotrizoaat (9%)	Apotheek AZL	LUMC_97902861
DNase I type IV	Sigma	Cat# D5025
HLA-A∗02 HCMV pp65_495-503_ (NLVPMVATV) peptide	LUMC	n.a.
PMA	Merck	Cat# P8139-1MG
Calcium Ionophore	Merck	Cat# C7522-1MG
Dasatinib	Merck	Cat# SML2589-50MG

**Critical commercial assays**		

eBioscience™ Foxp3/Transcription Factor Staining Buffer Set	eBioscience	Cat# 00-5523-00
Zombie Aqua™ Fixable Viability Kit	Thermofisher	Cat# 423101
BD Horizon™ Brilliant Stain Buffer	BD	Cat# 566349
PE/Cy7® Conjugation Kit - Lightning-Link®	Abcam	Cat# ab102903
PE/Cy5® Conjugation Kit - Lightning-Link®	Abcam	Cat# ab102893
PE/R-Phycoerythrin Conjugation Kit - Lightning-Link®	Abcam	Cat# ab102918
DyLight® 405 Conjugation Kit (Fast) - Lightning-Link®	Abcam	Cat# ab201798
DyLight® 488 Conjugation Kit (Fast) - Lightning-Link®	Abcam	Cat# ab201799
Alexa Fluor® 647 Conjugation Kit (Fast) - Lightning-Link®	Abcam	Cat# ab269823
APC/Cy7® Conjugation Kit - Lightning- Link®	Abcam	Cat# ab102859

**Software and algorithms**		

Aurora 5-laser	Cytek	-
SpectroFlo acquisition software V3	Cytek	-
Omiq	Dotmatics	https://www.omiq.ai/

**Other**		

UltraComp eBeads™ Plus Compensation Beads	Invitrogen	Cat# 01-3333-42
Corning® 96-well Clear Flat Bottom Polystyrene TC-treated Microplates, Individually Wrapped, with Lid, Sterile	Corning	Cat# 3599
Microplate, 96 well, ps, v-bottom, clear	Greiner Bio-one	Cat# 651101

## Materials and equipment

**Table:** FACS buffer – Store at 4°C for up to 1 month

*Reagent*	*Final Concentration*	*Amount*
PBS	N.A	500 mL
FCS	1%	5 mL
Sodium azide (10%)	0.02%	1 mL

**Table:** Complete medium – Store at 4°C for up to 1 month

*Reagent*	*Final Concentration*	*Amount*
IMDM	N.A	500 mL
Human Serum	8%	50 mL
Penicillin & Streptomycin	50 IU/mL & 50 μg/mL	3 mL
L-Glutamine	2 mM	5 mL

## Step-by-step method details

### Isolation of lymphocytes from buffy coats and human spleens

**Timing: 2 h**

This procedure describes the isolation of human peripheral blood mononuclear cells (PBMCs) from buffy coats or lymphocytes from spleen tissue under sterile conditions. Before starting, pre-cool PBS to 4°C.

1. Generate a single-cell suspension from buffy coat or spleen:
  a. Buffy coat: Transfer 20 mL buffy coat into a 50 mL tube and add 30 mL PBS.
  b. Spleen: Mechanically dissociate human spleen tissue (∼1 cm^3^) through a 70 μM cell strainer into a 50 mL tube. Add 30 mL cold PBS. After generating a single-cell suspension, process spleen-derived cells identically to PBMCs, including density gradient centrifugation and washing steps.

***Note:*** Take care to avoid spills, as human-derived materials should be treated as potentially hazardous.

2. Centrifuge the samples (300 × *g*, 15 min, 4°C). Carefully decant the supernatant.
3. Prepare density gradient tubes by adding 15 mL Ficoll to one 50 mL tube per donor.
4. Resuspend cell pellet in 30 mL PBS. Carefully layer the suspension on top of the Ficoll.

**CRITICAL:** Maintain a clearly visible interface between the cell suspension and Ficoll.

***Note:*** If the interface becomes disturbed, remove Ficoll by washing the cells 2–3 times with PBS (300 × *g*, 5 min, 4°C). Repeat Step 4 once Ficoll is fully removed.

5. Perform density-gradient separation by centrifugation (500 × *g*, 20 min, no brake, 4°C).

***Note:*** After centrifugation, three distinct layers and a cell pellet are visible. The middle layer, located at the plasma-Ficoll interface, appears as a white, cloudy ring and contains the lymphocytes (PBMC fraction). For more details on this matter, we would like to refer to Domínguez-Andrés *et al.*, 2021.^2^

6. Carefully collect the lymphocyte layer at the cellular interphase, and transfer it to a new 50 mL tube.
7. Wash the cells with PBS by filling the tube to the maximum volume and centrifuge (300 × *g*, 10 min, 4°C).
8. Carefully discard the supernatant, and resuspend cells in 10 mL complete medium.

***Note:*** Frozen PBMCs may be used if rested for at least 2 h and up to 16 h at 37°C with 5% CO_2_ in a T25 flask containing complete medium supplemented with 30 μg/mL DNAse I (type IV).

9. Count viable cells using a cell counter (e.g., Bürker-Türk counting chamber in combination with Trypan Blue to exclude dead cells, or an automated cell counter, which may also use Trypan Blue). Adjust the cell concentration to 10 × 10^6^ cells/mL.
10. Proceed based on the desired downstream analysis with ‘Preparation of unstimulated antigen-specific CD8^+^ T cells’ or ‘Preparation of peptide-stimulated antigen-specific CD8^+^ T cells’.

### Preparation of unstimulated antigen-specific CD8^+^ T cells

**Timing: 10 min**

This procedure describes the plating of unstimulated cells. The correct number of cells per well is critical and prevents biases between samples induced by differences in cell numbers. Use the appropriate single-channel or multichannel pipette according to the indicated volume.

11. For each sample, plate 1 × 10^6^ cells per well in a 96-well plate in 100 μl complete medium.

**CRITICAL:** Plate equal numbers of cells per well for accurate comparisons between samples, as differences in cell number can affect staining intensity.

***Note:*** A round-bottom (U-bottom) 96-well plate facilitates easier resuspension when working with large numbers of cells (≥1 × 10^6^), whereas a conical (V-bottom) 96-well plate promotes tighter pellet formation and is preferred for lower cell numbers (<1 × 10^6^) to minimizing cell loss during washing steps.

***Note:*** When comparing donors or multiple time points, include PBMC samples as a standard from a frozen reference batch, preferably in triplicate, to assess internal variation and enable normalization.

12. Prepare (single-stain) reference controls for spectral flow of antigen-specific CD8^+^ T cells:
  a. Plate 1 × 10^6^ cells in one well for the unstained reference control.
  b. Plate 1 × 10^6^ cells in one well for the live/dead single-stain reference control (see step 21).
  c. If using cells as single-stain reference controls (instead of beads), plate 1 × 10^6^ cells per well for each fluorophore. Use separate wells for surface staining (see step 23) and intracellular staining (see step 30).

***Note:*** Both beads and cells can be used for (single-stain) reference controls for spectral unmixing. It may be required to determine whether cells or antibody-binding beads are more appropriate for validating the antibodies used in your panel.

***Note:*** Plating cells for FMO controls are optional, as the reference controls provide sufficient information for unmixing, and well-defined positive/negative populations allow accurate gating.

### Preparation of peptide-stimulated antigen-specific CD8^+^ T cells

**Timing: 22 h incubation**

MHC class I tetramers may sometimes fail to detect antigen-specific CD8^+^ T cells, for example due to T cell receptor internalization after activation. In that case, CD137 upregulation can serve as a reliable surrogate marker for antigen-specific activation. In this protocol, we focus on human cytomegalovirus (HCMV)-specific CD8^+^ T cells stimulated with peptide and identified by CD137, as an example. The total hand-on time is 1.5–2 h.

13. For each sample, plate 1 × 10^6^ cells per well in triplicate in a 96-well U-bottom or flat-bottom plate with 100 μL complete medium per well. Each set of triplicates should include a negative control, a positive control, and a peptide-stimulated condition, see step 15).

**CRITICAL:** Plate equal numbers of cells per well for accurate comparisons between samples, as differences in cell number can affect staining intensity.

***Note:*** When comparing donors or multiple time points, include PBMC samples as a standard from a frozen reference batch, preferably in triplicate, to assess internal variation and enable normalization.

14. Prepare (single-stain) reference controls for spectral flow of antigen-specific CD8^+^ T cells:
  a. Plate 1 × 10^6^ cells in one well for the unstained reference control.
  b. Plate 1 × 10^6^ cells in one well for the live/dead single-stain reference control (see step 21).
  c. If using cells as single-stain reference controls (instead of beads), plate 1 × 10^6^ cells per well for each fluorophore. Use separate wells for surface staining (see step 23) and intracellular staining (see step 30).

***Note:*** Both compensation beads and cells can be used as (single-stain) reference controls for spectral unmixing. Compensation beads are particularly useful when the target protein is expressed at low levels or the cell population of interest is rare.

***Note:*** Plating cells for FMO controls are optional, as the reference controls provide sufficient information for unmixing, and well-defined positive/negative populations allow accurate gating.

15. For each set of triplicates plated in step 13, prepare the stimulation conditions as follows:
  a. Negative control: To the designated well of plated cells for each sample, add 100 μL of complete medium. Do not add any stimulants or peptides.
  b. Positive control - PMA/ionomycin stimulation: To the designated well of plated cells for each sample, add 100 μL of complete medium containing PMA (final concentration 25 ng/mL) and ionomycin (final concentration 0.25 μg/mL).
    i. Add to each well 100 μL of complete medium containing 0.5 μL of PMA (from 10 μg/mL working stock) and 0.5 μL of ionomycin (from 100 μg/mL working stock).
  c. Peptide stimulation: To the designated well of plated cells for each sample, add 100 μL of complete medium containing peptide to achieve a final peptide concentration of 5 μg/mL.
    i. E.g. for stimulation with HCMV pp65_495-503_ peptide (1 mg/mL stock): add 1 μL to 99 μL complete medium (5 μg/mL end concentration).

***Note:*** A peptide concentration of 1–10 μg/mL generally provides effective stimulation when using short peptides (8–11 amino acids). For longer peptides (15–35 amino acids), optimization may be required, although a range of 10–30 μg/mL is typically sufficient. Ensure that the cell suspension contains antigen-presenting cells to process long peptides for MHC class I presentation.

***Note:*** PMA/ionomycin activates all T cells as a positive control. Final concentration can be optimized in the range from 10–50 ng/mL for PMA or 0.25–1 μg/mL for calcium ionomycin to improve cell viability as long incubation times with high concentration leads to overstimulation and cell death. Alternatively, T cells can be stimulated with αCD3/αCD28, concanavalin A, or phytohemagglutinin.

16. Incubate the cells for 16–20 h at 37°C in a humidified incubator with 5% CO_2_.

***Note:*** Depending on the research question, shorter and longer incubation may be required. In general, increased expression of key (rate-limiting) metabolic enzymes can be detected approximately 12–144 h after T cell activation. In longer-term culture systems where proliferation rates may differ between conditions, cells should be re-counted prior to staining.

17. In case of flat-bottom plate usage, transfer the cells to a round-shaped (U-bottom) or conically-shaped (V-bottom) plate in preparation for downstream viability and surface staining.

### Viability and cell-surface flow cytometry staining

**Timing: 45 min**

After sample preparation, determine cell viability using a live/dead marker (zombie aqua), followed by cell surface staining to define T cell subsets (CD197, CD45RA), antigen-specific T cells (tetramer or CD137), and activated/proliferating T cells (e.g.,.CD25/Ki-67). Perform all washing steps at the indicated centrifugal force, and remove the supernatant carefully after each centrifugation step.

18. Centrifuge the plate (400 × *g*, 4 min, 4°C), and remove supernatant.
19. Block Fc receptors: Add 50 μL FcBlock and incubate for 10 min in the fridge.
  a. Prepare FcBlock by adding 2.5 μL Human TruStain FcX to 500 μL FACS buffer (sufficient for 10 samples).
20. Wash cells once by adding 150 μL ice-cold PBS to each well. Centrifuge the plate at 400 × *g* for 5 min at 4°C and remove supernatant.
21. Stain for viability: Add 50 μL of the prepared Live/Dead staining solution to each sample. Incubate for 15 min at 4°C, protected from light.
  a. Prepare Live/Dead stain by diluting the reagent 1:1000 in cold PBS (e.g., add 1 μL to 1000 μL cold PBS).
22. Wash cells once by adding 150–200 μL cold FACS buffer to each well. Centrifuge the plate at 400 × *g* for 4 min at 4°C and remove supernatant.
23. Prepare the antibody mix for cell surface staining:
  a. Use MHC class I tetramers for detection of antigen-specific CD8^+^ T cells in unstimulated/resting (Table 1) settings or.

**Table 1:** Surface staining with tetramer in unstimulated/resting cells

*Reagent*	*Final Concentration*	*Amount (per 10 samples, total volume: 300* μ*L)*
FACS buffer	46%	138.4 μL
Brilliant stain buffer	46%	138.4 μL
αCD8 – APC-Fire810	NA. (1:1600)	0.19 μL
αCD25 – BV711	NA. (1:200)	1.5 μL
αCD3 – BUV395	NA. (1:50)	3 μL
αCD45RA – BUV496	200 μg/mL (1:1600)	0.19 μL
αCD197 – BV785	NA. (1:50)	6 μL
HLA-A∗01 HCMV pp65_363–373_ (YSEHPTFTSQY) – APC (1 mg/mL)	0.2 μg/μL (1:50)	6 μL
HLA-A∗02 HCMV pp65_495–503_ (NLVPMVATV) – APC (1 mg/mL)	0.25 μg/μL (1:50)	6 μL
Dasatinib (50 μM in DMSO)	0.05 μM (1:1000)	0.3 μL
  b. Use CD137 as a surrogate marker following peptide stimulation (Table 2).

**Table 2:** Surface staining of cells for CD137 after peptide stimulation

*Reagent*	*Final Concentration*	*Amount (per 10 samples, total volume: 300* μ*L)*
FACS buffer	46%	138.4 μL
Brilliant stain buffer	46%	138.4 μL
αCD25 – BV711	NA. (1:200)	1.5 μL
αCD69 – Percp-Cy5.5	NA. (1:50)	3 μL
αCD38 – APC-Fire810	NA. (1:50)	6 μL
αCD137 – BV650	NA. (1:50)	6 μL
  c. Use recommended dilution per antibody for each single-stain reference control (Tables 1 and 2).

***Note:*** Dasatinib improves the detection of T cells with low-affinity TCR–pMHC interactions by preventing internalization of the TCR.^3^ Tetramer staining can be further improved by a pre-incubation of 15 min on 37^°^C with 50 nM dasatinib in FACS buffer.

***Note:*** Multiple tetramers can be used per staining to assess a broader range of tumor- or virus specific T cell clones.

24. Surface staining: Add 30 μL of the antibody/tetramer mix to each sample and incubate for 30 min at 4°C (in the dark).

***Note:*** Tetramer staining is often more efficient at room temperature, so adjust accordingly if tetramers are included.

25. Wash the cells twice with 150–200 μL cold FACS buffer per well. Centrifuge at 400 × *g* for 4 min at 4°C and remove supernatant after each centrifugation step.

### Intracellular staining for metabolic proteins

**Timing: 2 h**

This panel enables simultaneous detection of multiple metabolic pathways (Figure 1). Cells are washed between staining steps at the indicated centrifugation speed. Remove the supernatant carefully after each centrifugation step.

26. After the final wash, add 100 μL fixation buffer per well, and gently resuspend the cells.
  a. Prepare fixation buffer by adding 250 μL Fixation/Permeabilization (eBioscience FoxP3 Fixation kit) to 750 μL Perm Diluent (eBioscience FoxP3 Fixation kit).

***Note:*** The dilution factor of the Fixation/Permeabilization solution can range from 4× to 16×, depending on the intracellular target. Some targets require milder fixation for optimal staining efficiency.

27. Incubate the samples and (single-stain) reference controls for 45 min at 20°C in the dark.

***Note:*** Incubation time with fixation buffer may vary between 30–60 min.

28. Centrifuge the plate (500 × *g*, 5 min, 20°C), and remove supernatant. Then wash twice by adding 150-200 μL permeabilization buffer per well. Centrifuge the plate (500 × *g*, 5 min, 20°C) and remove supernatant after each centrifugation step.
  a. Prepare permeabilization buffer by adding 1 mL Perm buffer 10× (eBioscience FoxP3 Fixation kit) to 9 mL Milli-Q.

***Note:*** Increased centrifugation speed and duration help to prevent cell loss during washing steps after fixation and permeabilization.

29. Prepare antibody mix for intracellular staining of T cells, either *unstimulated/resting* (Table 3) or *peptide stimulation* (Table 4), according to the experimental design.

**Table 3:** Intracellular staining of unstimulated/resting cells

*Reagent*	*Final Concentration*	*Amount (per 10 samples, total volume: 300* μ*L)*
1x Perm buffer	98%	294 μL
αKi-67 – PE-Dazzle594	NA. (1:200)	1.5 μL
αGLUT1 – Dylight405	1.4 ng/μL (1:600)	0.5 μL
αPKM – PE	2.1 ng/μL (1:400)	0.75 μL
αG6PD – APC-Cy7	1.0 ng/μL (1:800)	0.5 μL
αCPT1a – PE-Cy5.5	1.4 ng/μL (1:600)	0.5 μL
αATP5a – FITC	1.4 ng/μL (1:600)	0.5 μL
αSDHA – AF647	0.42 ng/μL (1:2000)	0.15 μL
αGlutaminase – PE-Cy7	1.4 ng/μL (1:600)	0.5 μL

**Table 4:** Intracellular staining of cells after peptide stimulation

*Reagent*	*Final Concentration*	*Amount (per 10 samples, total volume: 300* μ*L)*
*1x Perm buffer*	*98%*	*294* μ*L*
*αCytochrome C – PE-Cy7*	*1.4 ng/μL (1:600)*	*0.5* μ*L*
*αCD98 – BUV395*	*200* μ*g/mL (1:400)*	*0.75* μ*L*
*αG6PD – APC-Cy7*	*1.0 ng/μL (1:800)*	*0.5* μ*L*
*αATP5a – FITC*	*1.4 ng/μL (1:600)*	*0.5* μ*L*
*αSDHA – AF647*	*0.42 ng/μL (1:2000)*	*0.15* μ*L*
30. Prepare single stain reference control:
  a. Use recommended dilution per antibody for each single-stain reference control (Tables 3 and 4).

***Note:*** Most metabolic targets (e.g., metabolic enzymes) are basally expressed in viable cells and are therefore expected to be positive in living cells. Be sure to include an unstained control sample for proper background and autofluorescence assessment.

31. After the final wash, add 30 μL antibody mix/single stain reference control to each sample and incubate for 30–45 min at 20°C.

***Note:*** A longer incubation time can improve signal intensity.

32. Wash the cells twice with 150 μL 1× permeabilization buffer per well. Centrifuge the plate at 500 × *g* for 5 min at 20°C and remove supernatant after each centrifugation step.
33. After the final wash resuspend each well in 100–150 μL FACS buffer for acquisition.

***Note:*** Before acquisition, transfer cells to the plates or tubes required by your flow cytometer, based on its sample-loading mode.

34. Create a mixed (single-stain) reference sample: Combine 10 μL of each single-stained reference control; additionally include 10 μL of the unstained control and 10 μL of the viability dye control, into a single tube to verify the accuracy of spectral unmixing.

***Note:*** Numerous metabolic antibodies are commercially available, however, many are not pre-conjugated to fluorophores. Several antibody labeling kits allow rapid conjugation of the antibody of interest within 3.5 h. Regardless of the method used, each newly generated antibody–fluorophore conjugate must be carefully titrated prior to experimental use to ensure optimal signal-to-noise performance.

### Acquisition and analysis

**Timing: 1.5h**

This panel was tested on a Cytek Aurora 5-laser spectral analyser and can be used on any spectral flow cytometer equipped with UV, Blue, Violet, Yellow-Green and Red lasers. Timing is based on 20 samples. Acquisition will take approximately two additional min per additional sample.

35. Run the start-up and quality control (QC) procedure of your spectral analyzer.
36. Open the experiment, adjust the forward scatter (FSC) and side scatter (SSC), and acquire the single-stained samples, unstained and live/dead controls.

***Note:*** Acquire a minimum of 50,000 events for cell samples and 5,000 events for bead controls to ensure reliable analysis in this experimental setup. As stated above, the number of events required for reliable analysis depends on the frequency of CD8^+^ T cells in your sample. Therefore, it is recommended to perform pilot experiments to determine the optimal number of events for your experimental setup.

37. After acquiring the single stained samples, perform spectral unmixing to apply the appropriate settings to your experiment.
38. Acquire the mixed single-stained sample and verify the unmixing.

***Note:*** The mixed single-stain sample is not biased by shared marker expression because each fluorochrome is represented independently, without co-expression effects that may occur in fully stained samples. Therefore, it can be used to refine or adjust unmixing matrix manually after acquisition.

39. Acquire the experimental samples and optional batch controls.
40. After acquisition, check the unmixing and make adjustments if necessary based on the mixed single-stain sample (Figure 2A).

**Figure 2:**
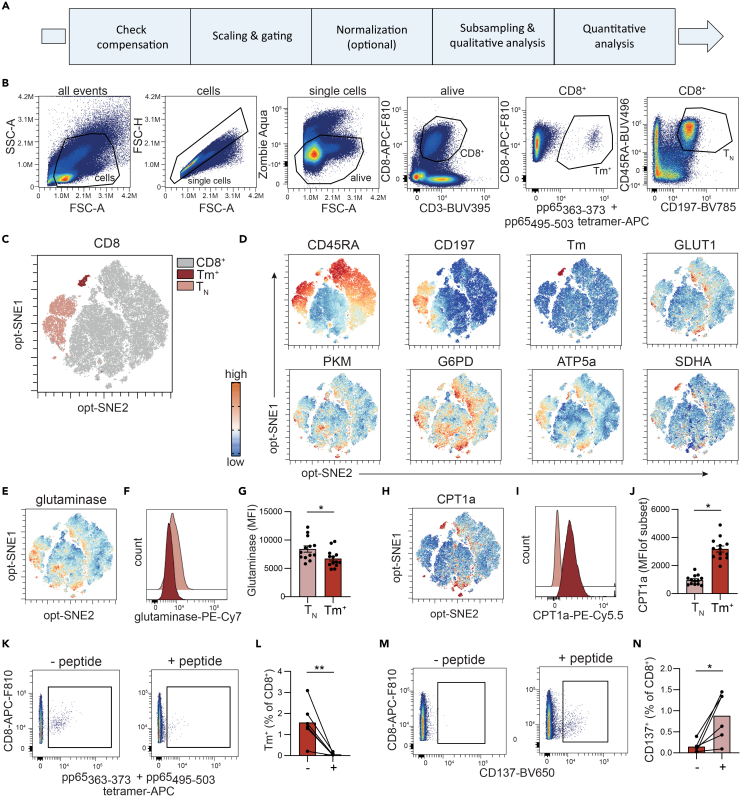
Representative workflow and results for metabolic profiling of rare CD8^+^ T cell populations (A) Schematic overview of the workflow following sample acquisition. (B) Gating strategy for viable CD8^+^ T cells, including naïve T cells (CD45RA^+^, CD197^+^) and antigen-specific T cells (Tetramer^+^) depicted as density plot. (C and D) opt-tSNE analysis including CD45RA, CD197, Tetramer pp65_363–373_ & pp65_495–503_, GLUT1, PKM, G6PD, ATP5a, SDHA, Glutaminase, CPT1a, CD28, CD25. C: Naïve and tetramer^+^ antigen-specific CD8^+^ T cells are indicated on the map. D: Relative expression of metabolic markers and T cell identifiers (blue: low expression; red: high expression). (E–J) Expression of glutaminase (E–G) and CPT1a (H–J) on indicated subsets depicted as opt-tSNE (E, H), histogram (F, I) and bars graphs (G, J). Whiskers represent mean ± SEM; dots represent individual donors. (K) Representative dot plot of PBMC-derived CD8^+^ T cell treated with and without peptide and stained with tetramers. (L) Quantification of tetramer^+^ T cells in samples with and without peptide stimulation. Bars graphs show mean ± SEM; dots represent individual donors. (M) Representative dot plot of PBMC-derived CD8^+^ T cells treated with peptide showing CD137 expression (N) Quantification of CD137^+^ T cells in samples with and without peptide stimulation. Bars graphs show means ± SEM; dots represent individual donors. Data in panels G, J, L and N were analyzed using two-sided paired *t* test. ∗*p* < 0.05, ∗∗*p* < 0.01, ∗∗∗*p* < 0.001, ∗∗∗∗*p* < 0.0001.
41. Apply appropriate gating and adjust scales to optimize population separation (Figure 2B).
42. Perform further downstream analysis (e.g., opt-tSNE, Figures 2C–2J) according to your research question.

## Expected outcomes

Each T cell subset has distinct metabolic preferences based on their location and/or metabolic needs. The metabolic markers included in this workflow survey core pathways central to T cell metabolic fitness, providing a broad view of the metabolic state of these T cells, e.g., naïve human T cells (CD45RA^+^CD197(CCR7)^+^) and antigen-specific memory T cells (detected by MHC class tetramers).^4^ As an example, the mean fluorescent intensity (MFI) of glutaminase and CPT1a was plotted for both naïve T cells and tetramer^+^ antigen-specific CD8^+^ T cells (Figures 2E–2J). These data show that antigen-specific CD8^+^ T cells have a significant decreased expression of glutaminase but increased expression of CPT1a compared to naïve T cells. These CCR7^-^ antigen-specific T cells have a memory phenotype, and previous studies have shown that this cell type relies on fatty acids.^4^

Activated antigen-specific CD8^+^ T cells can be identified using a similar workflow, including αCD137. Upon peptide stimulation mimicking cognate antigen, activated CD8^+^ T cells upregulate CD137, while the T cell receptor is downregulated resulting in decreased detection by tetramers (Figures 1K–1N). Subsequently, the expression of key metabolic markers can be analyzed on CD137^+^ CD8^+^ T cells, as shown in Figure 3 of Mülling *et al.*^1^^,^^5^ Panels may be extended to interrogate additional metabolic pathways such as fatty acid synthesis (e.g., fatty acid synthetase (FASN)) or cholesterol biosynthesis (e.g., Farnesyl-Diphosphate Farnesyltransferase 1 (FDFT1)), with further examples provided in *Mülling et al.*^1^Although protein abundance is not a direct measure of metabolite flux, the expression of these metabolic regulators closely reflects functional metabolic activity.^6^

## Quantification and statistical analysis

Before analyzing acquired data, verify that spectral unmixing is correct by inspecting a matrix plot of the mixed single-stained sample. If data are acquired on multiple days or instruments, normalize using a, preferably in triplicate, batch control and tools such as CytoNORM.^7^ To visualize the metabolic landscape of antigen-specific T cells, perform dimensionality reduction analysis using opt-tSNE (Figure 2).^8^ For quantitative analyses, gate subsets of interest (e.g., antigen-specific CD8^+^ T cells, as shown in Figure 2) and extract mean fluorescence metrics. Because metabolic markers typically manifest as gradual shifts in fluorescence intensity rather than discrete positive/negative populations, binary gating is usually not suitable (Figures 2K–2N). Therefore, (geometric) mean fluorescence intensity (MFI) is recommended as the primary quantitative measure of metabolic protein abundance. Testing of significance is dependent on the research question. In general, comparisons between two groups are analyzed using two-sided unpaired *t* test between different T cell subsets and two-sided paired *t* test for stimulated *versus* non-stimulated cells. For comparisons across more than two groups, repeated measures ANOVA with Tukey’s multiple comparisons test was used. Normality was assumed based on prior validation of this experimental system, and parametric tests (two-side t-tests, ANOVA) were used accordingly. Significance: *p*-values <0.05 are considered significant and are indicated as follows: ∗*p* < 0.05, ∗∗*p* < 0.01, ∗∗∗*p* < 0.001, ∗∗∗∗*p* < 0.0001.

## Limitations

Flow cytometry–based metabolic profiling provides pathway-specific resolution but is inherently constrained by the choice and availability of antibodies. Global approaches, including transcriptomics and metabolomics, offer broader coverage but are often limited by low cell numbers, particularly for rare antigen-specific T cell populations. Emerging single-cell omic techniques, including the spectral flow cytometry-based approach described here, may help overcome these limitations. However, accurate interpretation of results requires strict standardization of experimental variables, including cell counting, viability assessment, reagent concentrations, and incubation conditions, to ensure that measured differences reflect genuine biological variation rather than technical artifacts.

## Troubleshooting

### Problem 1

Tetramer^+^ antigen-specific CD8^+^ T cells are below thresholds or not detected in the sample.

### Potential solution

*Low precursor frequency* – Antigen-specific CD8^+^ T cells may be very rare. Consider pooling samples, enriching for CD8^+^ T cells, or increasing total cell input. If pooling is performed, adjust the number of wells accordingly at this stage.

• *Cell viability* – Dead or stressed cells may fail to stain or may give high background signal. Assess viability and optimize handling to minimize stress.
• *Fluorophore or instrument issues* – Ensure the spectral flow cytometer is properly configurated, unmixing has been correctly applied, and that the fluorophores to detect antigen-specific CD8^+^ T cells are compatible with the panel.

### Problem 2

Lack of detecting CD137 upregulation on activated CD8^+^ T cells compared to the unstimulated controls.

### Potential solution

• *Insufficient stimulation* – For activation marker-based detection, ensure appropriate antigen concentration and incubation time. Include positive controls, such as PMA/ionomycin or αCD3/αCD28, to confirm detection sensitivity.

### Problem 3

Fluorescent metabolic markers show weak or undetectable signals.

### Potential solution

• *Too high cell number per sample* –Antibody concentrations used here are based on 0.5 million to 2.5 million cells per sample. Either lower your number of cells per sample or increase staining volume or titrate antibody for a different number of cells.
• *Autofluorescence* - Autofluorescence due to different cell consistencies per tissue may interfere. To solve this issue, unmix per cellular source or extract autofluorescence manually.
• *Spectral overlap* - Spectral overlap can distort fluorescence signals and reduce apparent marker expression. If spectral overlap is excessive, consider replacing one or more fluorophores to minimize interference. Titrate all antibodies to achieve optimal resolution and use fluorescence minus one (FMO) controls to optimize panels. Once the spectral flow panel is optimized, FMO controls are optional, as single-stain reference controls provide the necessary information for spectral unmixing and allow accurate gating.
• *Inaccurate unmixing* – Incorrect definition of the positive single-stain reference population can result in inaccurate spectral unmixing. Ensure that each single-stain control contains a clearly defined positive population and verify proper reference selection before performing unmixing. If necessary, refine the unmixing matrix by updating the reference controls or performing manual adjustment of the unmixing parameters.

### Problem 4

Observed metabolic profiles and/or frequencies of antigen-specific T cells vary widely between donors.

### Potential solution

• *Biological heterogeneity* – Differences may reflect actual biological variation due to factors such as age, sex, infection history, or immune status, all of which can influence T cell metabolism. Including multiple donors helps capture this variability, and stratifying donors based on these parameters may further reduce observed variation.
• *Sample handling and processing* – Variations in collection, transport, or processing can affect both cell viability and metabolic activity. Standardizing sample collection and handling helps minimize technical variation. Specifically, using similar cell numbers across samples is essential. When samples are acquired on multiple days, include an internal standard for data normalization.^7^^,^^9^

## Resource availability

### Lead contact

Further information and requests for resources and reagents should be directed to and will be fulfilled by the lead contact, Ramon Arens (r.arens@lumc.nl).

### Technical contact

Technical questions on executing this protocol should be directed to and will be answered by the technical contact, J. Fréderique de Graaf (j.f.de_graaf@lumc.nl).

### Materials availability

This protocol did not generate new unique reagents.

### Data and code availability

This protocol did not generate or analyze datasets or codes.

## Acknowledgments

The authors thank the LUMC Flow Cytometry Core Facility for their support and assistance with the operation of spectral flow cytometry and Hanna Hepp, Shengchun Cai, and Ward Vleeshouwers for their feedback on the manuscript. N.M. received personal research funding from 10.13039/501100004426Dr. Werner Jackstädt-Stiftung (project no. S0134–10.124) and the 10.13039/501100001659German Research Foundation (project no. 526085745). J.F.d.G. received personal research funding from the Leids Universitair Fonds (project no. LUF25290), and R.A. received personal research funding from the 10.13039/501100003246Dutch Research Council (10.13039/501100003246NWO) Domain Science (Open Competition ENW.XS25.2.129).

## Author contributions

Conceptualization, J.F.d.G. and R.A.; methodology, J.F.d.G., N.M., and R.A.; performing experiments, J.F.d.G. and N.M.; formal analysis, J.F.d.G. and N.M.; visualization, J.F.d.G. and R.A.; writing – original draft, J.F.d.G. and R.A.; review and editing, N.M. All authors read and approved the final manuscript.

## Declaration of interests

The authors declare no competing interests.

## References

[1] Mülling N., de Graaf J.F., Heieis G.A., Boss K., Wilde B., Everts B., Arens R. (2025). Metabolic profiling of antigen-specific CD8^+^ T cells by spectral flow cytometry. Cell Rep. Methods.

[2] Domínguez-Andrés J., Arts R.J.W., Bekkering S., Bahrar H., Blok B.A., de Bree L.C.J., Bruno M., Bulut Ö., Debisarun P.A., Dijkstra H. (2021). *In vitro* induction of trained immunity in adherent human monocytes. STAR Protoc..

[3] Lissina A., Ladell K., Skowera A., Clement M., Edwards E., Seggewiss R., van den Berg H.A., Gostick E., Gallagher K., Jones E. (2009). Protein kinase inhibitors substantially improve the physical detection of T-cells with peptide-MHC tetramers. J. Immunol. Methods.

[4] Corrado M., Pearce E.L. (2022). Targeting memory T cell metabolism to improve immunity. J. Clin. Investig..

[5] Mülling N., Behr F.M., Heieis G.A., Boss K., van Duikeren S., van Haften F.J., Pardieck I.N., van der Gracht E.T.I., Vleeshouwers W., van der Sluis T.C. (2024). Inhibiting the NADase CD38 improves cytomegalovirus-specific CD8+ T cell functionality and metabolism. J. Clin. Investig..

[6] Hartmann F.J., Mrdjen D., McCaffrey E., Glass D.R., Greenwald N.F., Bharadwaj A., Khair Z., Verberk S.G.S., Baranski A., Baskar R. (2021). Single-cell metabolic profiling of human cytotoxic T cells. Nat. Biotechnol..

[7] Van Gassen S., Gaudilliere B., Angst M.S., Saeys Y., Aghaeepour N. (2020). CytoNorm: A Normalization Algorithm for Cytometry Data. Cytometry. A..

[8] Belkina A.C., Ciccolella C.O., Anno R., Halpert R., Spidlen J., Snyder-Cappione J.E. (2019). Automated optimized parameters for T-distributed stochastic neighbor embedding improve visualization and analysis of large datasets. Nat. Commun..

[9] Liechti T., Van Gassen S., Beddall M., Ballard R., Iftikhar Y., Du R., Venkataraman T., Novak D., Mangino M., Perfetto S. (2023). A robust pipeline for high-content, high-throughput immunophenotyping reveals age- and genetics-dependent changes in blood leukocytes. Cell Rep. Methods.

